# Approach to abnormal uterine bleeding in presence of endometrial polyps with new hysteroscopic devices

**DOI:** 10.1007/s00404-025-08005-7

**Published:** 2025-03-29

**Authors:** Francesco G. Martire, Giorgia Schettini, Eugenia Costantini, Claudia d’Abate, Giuseppe Sorrenti, Gabriele Centini, Errico Zupi, Lucia Lazzeri

**Affiliations:** 1 Department of Molecular and Developmental Medicine, Obstetrics and Gynecological Clinic, University of Siena, Strada Delle Scotte 14, 53100 Siena, Italy; 2 Department of Gynecology, San Carlo Nancy Hospital, Rome, Italy

**Keywords:** Abnormal uterine bleeding, Endometrial polyp, Hysteroscopy, Morcellator, See-and-treat hysteroscopy

## Abstract

**Purpose:**

To compare different endoscopic techniques, such as hysteroscopy with morcellator and traditional resectoscopy, and different surgical settings, such as operating room setting and outpatient setting, for patients with abnormal uterine bleeding (AUB) and suspected endometrial polyps.

**Metho:**

In this prospective study, 180 women diagnosed with endometrial polyps on ultrasound were enrolled. Patients were divided into three groups: 1) resectoscopy under anesthesia in an operating room setting; 2) morcellation with anesthesia in an operating room setting; and 3) outpatient morcellation without anesthesia. The main outcomes included procedure completion rates, operative time, patient satisfaction, and pain intensity using the Visual Analog Scale (VAS). Additionally, histological analysis was conducted for all cases.

**Results:**

Among the 180 patients, all procedures were completed in Groups 1 and 2, while Group 3 had a 96.7% completion rate. Procedure duration was the shortest in Group 3 (average 6.5 min), significantly less than in Group 1 (*p* value < 0.05; CI 95%). Pain was manageable in all groups, with VAS scores < 4 for most patients in the outpatient setting. Histology confirmed benign polyps in most cases, and malignant or premalignant conditions were around 3% of procedure.

**Conclusion:**

Outpatient “see-and-treat” hysteroscopy with morcellator, performed without anesthesia, proved feasible, safe, and cost-effective, with minimal discomfort and comparable diagnostic accuracy to traditional methods. This technique offers a practical approach for the management of AUB, enabling efficient treatment while reducing costs and resource usage, and may be considered as a preferred option in appropriate patients.

## What does this study add to the clinical work

**Table:** 

The main aim of the study is to demonstrate the feasibility and non-inferiority of in-office ‘see and treat’ hysteroscopy for the treatment of intrauterine pathologies compared to traditional methods under sedation. In addition, it's possible to note the reduction in operating time and number of hospital visits by patients and all that this entails in economic terms.	

## Introduction

Abnormal uterine bleeding (AUB) is a common reason women seek gynecological care, accounting for up to 15% of office visits and 25% of gynecological surgeries. It can affect up to 30% of women at any stage of reproductive life, from puberty to menopause [1]. The International Federation of Gynecology and Obstetrics (FIGO) developed the PALM-COEIN classification system to categorize AUB causes into structural factors (PALM: Polyp, Adenomyosis, Leiomyoma, Malignancy/Hyperplasia) and non-structural factors (COEIN: Coagulopathy, Ovulatory dysfunction, Endometrial, Iatrogenic, and Not otherwise classified) [2]. Endometrial polyps, classified as "P" within the PALM acronym, consist of endometrial glands, stroma, and blood vessels and are characterized localized overgrowths of endometrial tissue within the uterine cavity, often associated with hormonal imbalances, particularly an excess of estrogen relative to progesterone [3, 4].

The incidence of endometrial polyps increases with age, especially among perimenopausal and postmenopausal women, with a prevalence of up to 10% among women of reproductive age and as high as 25% in those over 40. While most endometrial polyps are benign and asymptomatic, histological evaluation is crucial to rule out hyperplasia, fibroids, sarcoma, and carcinoma, particularly in older women. In presence of risk factors, such as symptoms (AUB), a polyp size > 10 mm or postmenopausal age, the prevalence of malignancy increases up to 4.9% [5, 6]. Differently, small endometrial polyps in premenopausal age may regress in up to 25–30% of cases in 12–18 months [7, 8]. Therefore, a correct diagnostic ITER and eventually an appropriate removal procedure is fundamental in patients with risk factors. Hysteroscopy is a key tool in the evaluation and management of abnormal uterine bleeding, which may be an indicator of an underlying oncological pathology as it allows a direct view of the uterine cavity. In addition, it offers an accurate diagnosis, reducing the need for invasive interventions and improving patients' quality of life. Ongoing studies keep exploring the relationship between polypectomy and fertility, suggesting potential benefits for those undergoing assisted reproductive techniques. In these patients, it is essential to conduct the most precise evaluation of the myometrium and endometrial cavity using minimally invasive approach, such as transvaginal ultrasound or hysteroscopy, to reduce the risk of failures with the chosen treatment approach [9].

For symptomatic cases, surgical polypectomy is preferred to reduce malignancy risk, improve quality of life and increase pregnancy rate in infertile women [10]. This is typically performed via hysteroscopy, a procedure that allows precise removal with minimal impact on surrounding tissue. Current hysteroscopic polypectomy methods include mechanical excision, electrosurgical techniques, and intrauterine morcellation, providing flexibility and efficiency in polyp management. Nowadays, an outpatient setting for hysteroscopic polypectomy with local or no anesthesia is possible due to miniaturized instruments that maintain comparable outcomes to those achieved in the operating room [1–11].

In cases of oncological risk factors or infertility concerns, polypectomy becomes a priority. Like all surgical procedures involve risks that vary according to the technique [12], including potential anesthesia complications when deep sedation is required [13], allowing a safe and fast approach, without reducing patient compliance, is the main objective of the development of new surgical devices.

This prospective study aimed to evaluate and compare two endoscopic techniques, hysteroscopy with a morcellator versus traditional resectoscopy, and two settings: operating room versus outpatient setting for treating endometrial polyps in patients with AUB.

## Methods

We conducted a prospective comparative study to analyze three groups of patients with AUB and endometrial pathology in particular suspected endometrial polyp. This study was conducted on 250 women diagnosed with AUB evaluated between 30 November 2022 and 30 June 2024. It was approved by the local Institutional Review Board (IRB) (registrar number 0002959/22).

In women of reproductive age, AUB was defined as bleeding that was abnormal in duration, quantity, frequency or regularity. For postmenopausal women, AUB was any uterine bleeding not associated with hormone therapy.

The selected patients were all over 18 years of age and were divided into three groups according to their treatment modalities, namely day-hospital operative hysteroscopy with the aid of anesthesia using a resectoscope or morcellator and outpatient hysteroscopy without anesthesia, with simultaneous polypectomy.

The main aim of the study is to demonstrate the feasibility and the non-inferiority of in-office ‘see-and-treat’ hysteroscopy for the treatment of intrauterine pathologies compared to traditional methods under sedation. Second, this study aimed to evaluate the efficacy of each procedure in terms of complete lesion removal, surgical time, histopathological outcomes, complications, patient compliance with procedures, and costs of office-based hysteroscopy without sedation compared to hysteroscopy with morcellator or resectoscope in day hospital.

Exclusion criteria are age under 18 years, women with ongoing pregnancy, women suspected of myometrial pathology (types 0, 1, and 2 per FIGO classification) and women without a detailed medical history.

**Fig. 1:**
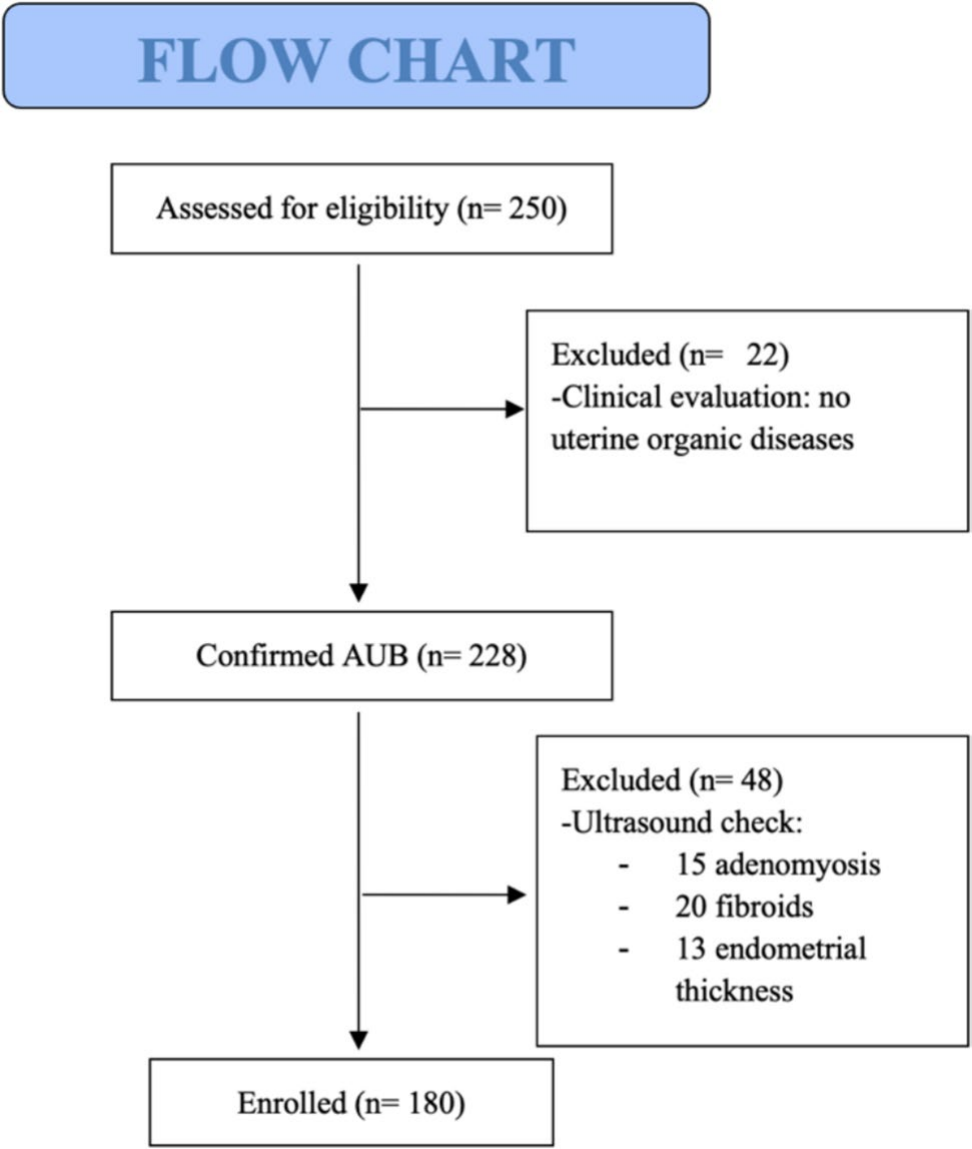
CONSORT flow diagram

Of the 250 women, 180 met the inclusion criteria, i.e., age, suspected endometrial polyp and treatment modality described above (Fig. 1).

They were, therefore, divided into three different groups: 1) patients undergoing diagnostic hysteroscopy followed by resectoscopic polypectomy under sedation in a day-hospital regimen, with a total of three visits performed; 2) patients undergoing diagnostic hysteroscopy followed by polypectomy by morcellation under sedation in a day-hospital regimen, with a total of three visits performed; 3) patients undergoing outpatient diagnostic hysteroscopy and concomitant polypectomy in a ‘see and treat’ approach, with a total of a single visit performed. When a patient is offered an office hysteroscopy, careful counseling is performed about the modalities of the procedure and the characteristics of the pain the woman may experience.

Three visits comprise outpatient access where the diagnostic question is posed, pre-hospital access comprising blood chemistry tests, electrocardiogram and subsequent anesthesiologic examination, and final day-hospital access to perform the operative hysteroscopy.

We evaluated treatment efficacy, surgical time, histopathological outcomes, pain using the visual analog scale (VAS) and all perioperative and postoperative complications in all groups.

Comprehensive medical histories were collected, including clinical, surgical, and obstetric data, such as age, body mass index (BMI), last menstruation/menopause/hormonal therapy, previous pregnancies, prior gynecological surgeries, and relevant comorbidities (hypertension, diabetes, dyslipidemia).

**Fig. 2:**
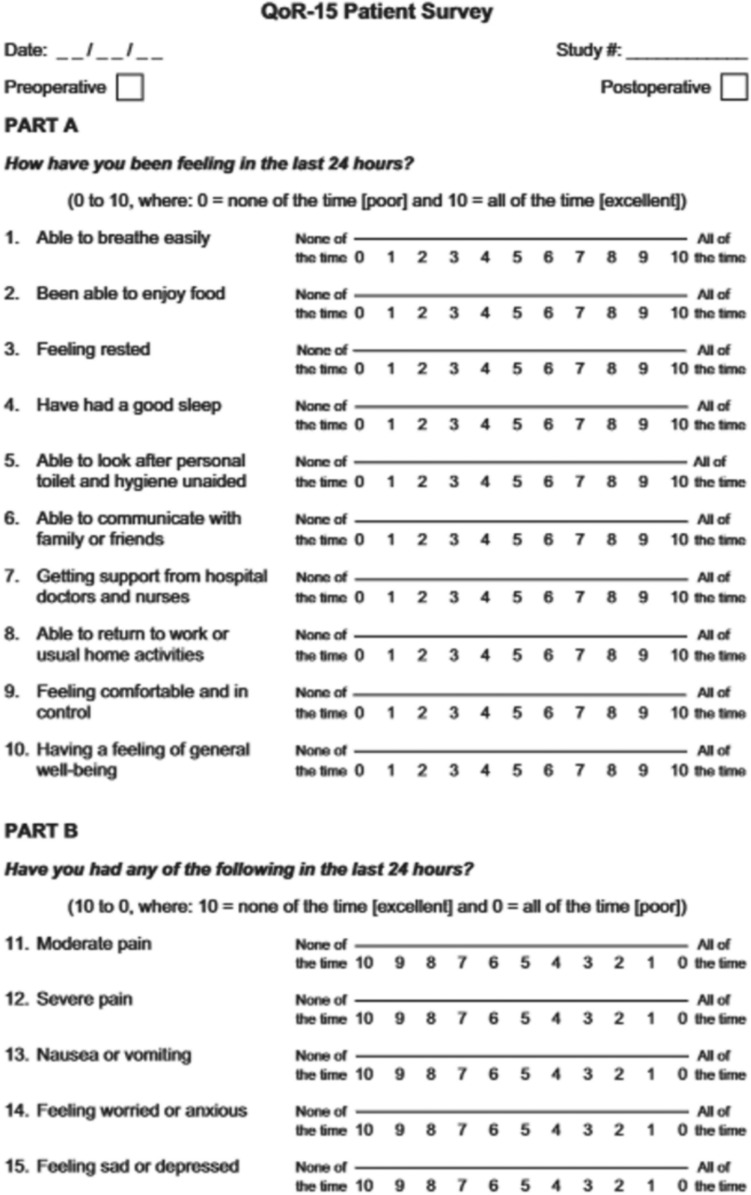
QoR-15 Patient Survey

For Group 1 e 2, we utilized the CRF RES-SHAVE questionnaire for medical history and administered the modified QoR-15 questionnaire to assess pain, nausea or vomiting related to anesthesia, and patient anxiety (Fig. 2).

Informed consent was obtained from all participants prior to transvaginal ultrasound and hysteroscopy. The authors declare no conflicts of interest.

Transvaginal ultrasound was performed using a 2D grayscale with Doppler to initially examine the uterine cavity, followed by a 3D assessment to provide a detailed morphological evaluation. The IETA (International Endometrial Tumor Analysis) criteria were applied to standardize the assessment of endometrial features, enhancing the accuracy of diagnosis. Key parameters included endometrial thickness, measured in the sagittal plane, and echogenicity, categorized as uniform or non-uniform according to the IETA recommendations. Additionally, vascularization was assessed with a color Doppler score, as per IETA guidelines, to evaluate blood flow characteristics within the endometrium. This score included specific parameters, such as vessel density, distribution, and pattern to assess abnormal angiogenesis, which can aid in distinguishing benign from malignant pathologies [14, 15].

Patients in Groups 1 and 2 underwent diagnostic hysteroscopy, followed by therapeutic hysteroscopy. In Group 1, bipolar resectoscopic hysteroscopy was performed for polypectomy, the instrument used has a diameter of 9 mm, direction of view 0 degrees, and required cervical dilatation by Hegar in each patient (Karl Storz, Tuttlingen, Germany). Group 2 utilized the Integrated Bigatti Shaver (Karl Storz, Tuttlingen, Germany) under sedation with saline solution as the distension medium. In this case, the instrument used has a diameter of 6 mm, direction of view 6 degrees, and consequently cervical dilatation was not necessary. Both groups received sedation with propofol and intraoperative analgesia with paracetamol (1000 mg).

In Group 3, diagnostic and therapeutic hysteroscopy were performed simultaneously without anesthesia, using the same Integrated Bigatti Shaver for polypectomy.

Surgical duration was recorded from instrument insertion to removal, and pain was assessed using the VAS at 1- hour post-surgery for Groups 1 and 2, while Group 3’s VAS score was averaged during the procedure and at 1-h post-surgery. We monitored perioperative and postoperative complications in each group and sent endometrial lesions for histological evaluation to confirm non-neoplastic status. We also compared the quantity of pathological material obtained across groups to identify differences between procedures.

Statistical analyses were performed using the SPSS v.15.0 (SPSS, Inc., Chicago, IL, USA). Continuous variables are reported as mean ± standard deviation (SD). Categorical variables are reported as a frequency or percentage. The statistical analyses initially assessed patient characteristics. Thereafter, the characteristics of hysteroscopy procedures and histological findings were evaluated in terms of percentage. Intergroup comparisons were performed using chi-square tests for categorical variables and independent sample t tests for continuous data. Fisher’s exact test was used to compare prevalence. Results with *p* < 0.05 were considered statistically significant.

We performed a statistical analysis to determine the sample size with significance level set at 95% and study power at 80% necessary to validate hypotheses. In order to validate this assumption, it was necessary to have a sample size of approximately 45 persons per group. Following the determination of the sample size to validate hypothesis, we collected data on 60 patients per group, i.e., by increasing the required sample size by 25% to cover any missing data and/or missing responses that could not be used for testing purposes.

## Results

Patients across groups were similar in age, BMI, menopausal status, and presence of comorbidities, ensuring a comparable baseline for analysis. Postmenopausal women represented the majority in Groups 1 and 3, while Group 2 had a higher proportion of premenopausal patients. BMI was slightly higher in Group 3. All the patients’ characteristics are shown in Table 1.

**Table 1:** Patient characteristics of Groups 1, 2, and 3

Patient characteristics	Group 1% (*n*) Mean ± SD	Group 2% (*n*) Mean ± SD	Group 3% (*n*) Mean ± SD	*P*-value
Age (years)	52.6 ± 11.7	46.2 ± 9.8	49.7 ± 5.2	> 0.05
Premenopausal	43.3% (26)	63.3% (38)	40.0% (24)	> 0.05
Postmenopausal	56.7% (34)	36.7% (22)	60.0% (36)	> 0.05
BMI	23.1 ± 3.4	23.0 ± 4.4	27.8 ± 2.1	> 0.05
Pregnancy history	2.2 ± 0.6	2.0 ± 0.7	2.3 ± 0.8	> 0.05
Nulliparity	36.7% (22)	23.3% (14)	20.0% (12)	> 0.05
Hypertension	26.7% (16)	30.0% (18)	16.7% (10)	> 0.05
Diabetes	13.3% (8)	6.7% (4)	3.3% (2)	> 0.05
Dyslipidemia	3.3% (2)	16.6% (10)	6.7% (2)	> 0.05
Prior surgery	36.7% (22)	46.6% (28)	13.3% (8)	> 0.05
Hormone therapy	6.7% (4)	0	13.3% (8)	> 0.05

The characteristics of the hysteroscopic procedures used in different groups are shown in Table 2. The procedures were completed successfully in all patients of Groups 1 and 2, while two patients in Group 3 experienced an incomplete procedure due to cervical stenosis, yielding a 96.7% completion rate. About the procedure duration, the shortest average duration was in Group 3, where morcellation was completed in 6.5 ± 1.6 min, significantly lower than both Group 1 (13.1 ± 5.0 min) and Group 2 (9.0 ± 3.5 min) (p < 0.05). Furthermore, in terms of pain and satisfaction, group 3 is not statistically inferior to Groups 1 and 2, recorded low VAS pain scores at 1 h post-procedure (2.0 ± 0.7) and high patient satisfaction (93.3%). Pain management in the outpatient setting without sedoanalgesia was adequate and comparable to anesthesia-supported procedures, indicating the feasibility of outpatient morcellation for patients tolerant of mild discomfort. Finally, Group 3 had minor surgical complications in 3.3% of cases, in particular a case of suspected endometritis manifested with fever and pelvic pain and a case of intraoperative bleeding for which treatment with tranexamic acid was necessary, while Group 2 experienced two case of anesthetic complication, which prolonged the operative time (14 min) and resulted in a higher-than-average VAS score (VAS 5), specifically a case of edema of the glottis and an episode of persistent nausea and vomiting. No surgical complications were observed in Group 1.

**Table 2:** Characteristics of the hysteroscopic procedure, pain symptom evaluation, and patient satisfaction rate (NS: not significant)

Hysteroscopic procedure	Group 1% (*n*) Mean ± SD	Group 2% (*n*) Mean ± SD	Group 3% (*n*) Mean ± SD	*P*-value
Completed	100% (60/60)	100% (60/60)	96.7% (58/60)	> 0.05
Incomplete	0.0% (0/60)	0.0% (0/60)	3.3% (2/60)	> 0.05
Pain (VAS) at 1 h	2.3 ± 0.8	2.7 ± 1.5	2.0 ± 0.7	> 0.05
Procedure duration (min)	13.1 ± 5.0	9.0 ± 3.5	6.5 ± 1.6	< 0.05
Patient satisfaction	100% (60/60)	93.3% (56/60)	93.3% (56/60)	> 0.05
Surgical complications	0.0% (0/60)	0.0% (0/60)	3.3% (2/60)	> 0.05
Anesthetic complications	0.0% (0/60)	3.3% (2/60)	0.0% (0/60)	> 0.05

In Table 3, the histological evaluation is shown. Histological examination confirmed benign endometrial polyps in most cases across groups (90% in Group 1, 86.7% in Group 2, and 83.3% in Group 3). Rates of atypical hyperplasia and carcinoma were consistent, reinforcing the diagnostic sufficiency of the outpatient approach for assessing endometrial pathology. The characteristics of uterine pathology are represented in Table 3. Fisher's test showed no statistically significant differences between the groups.

**Table 3:** Uterine pathology characteristics

Uterine pathology	Group 1 *n* (%)	Group 2 *n* (%)	Group 3 *n* (%)	*P*-value
Benign endometrial polyp	54 (90%)	52 (86.7%)	50 (83.3%)	> 0.05
Endometrial thickening	4 (6.7%)	2 (3.3%)	4 (6.7%)	> 0.05
Atypical endometrial hyperplasia	0	2 (3.3%)	4 (6.7%)	> 0.05
Endometrial carcinoma	0	2 (3.3%)	2 (3.3%)	> 0.05
Insufficient for evaluation	2 (3.3%)	2 (3.3%)	0	> 0.05

Moreover, all our patients were followed up with ultrasound at 6 months after hysteroscopy and in none of them, irrespective of the group they belonged to and therefore of the method used, did endometrial polyp recur; further highlighting the overlapping efficacy of the methods examined.

## Discussion

This study evaluates different hysteroscopic techniques and surgical settings for treating symptomatic endometrial polyps, focusing on both safety and efficiency. This study demonstrates that outpatient morcellation (Group 3) is non-inferior to resectoscopy under anesthesia (Group 1) and morcellation under anesthesia (Group 2) in terms of efficacy in treating endometrial polyps. Using smaller instrument, outpatient morcellation minimizes patients’ discomfort and pain, allowing for simultaneous diagnosis and treatment in a single procedure. [16] This approach significantly reduces operative time, eliminating the need for repeated insertion and removal of the hysteroscope through the cervix multiple times. For "see-and-treat" patients, procedure times averaged 6.5 ± 1.6 min, compared to 13.13 ± 5.02 min in traditional resectoscopic procedures. The ability to diagnose and treat endometrial pathology during a single outpatient visit enhances patient compliance and reduces the number of necessary medical appointments.

All surgical procedures carry inherent risks, and hysteroscopic interventions are no exception. [11, 12] It is therefore crucial to perform any type of surgical treatment only when there is a clear medical indication. Within various surgical options available, each technique has its own set of advantages and disadvantages. As such, it is equally important to select the most appropriate approach for each patient, minimizing potential risks while maximizing therapeutic benefits [17].

The choice of surgical technique, including the need for anesthesia, should be individualized to each patient's needs to balance safety with therapeutic efficacy. Considering healthcare costs, waiting times, and patient expectations is also essential when planning treatment [18].

The use of morcellation in an outpatient setting brings forward the advantage of reduced anesthetic risks. In settings where deep sedation is necessary, performing the procedure in a controlled operating room allows for better pain and anxiety management, with anesthesia personnel present to address any adverse reactions. However, deep sedation carries specific risks, such as respiratory depression, hypotension, or the potential for aspiration, all of which require vigilant monitoring. [19, 20] The use of local anesthesia could be a valid solution to reduce algic symptoms in patients who are anesthesiologically complex, who present contraindications to deep sedation, or who would like to avoid treatment in the operating room but who have clinical features deserving longer hysteroscopic times, as well as patients with a low tolerance threshold. However, discomfort caused using the speculum would be unavoidable. Instead, pain control strategies can be used for better management of office hysteroscopy, e.g., using topical anesthetic gels or taking NSAIDs pre-procedure.

Furthermore, the morcellator technique also presents several safety benefits over traditional resectoscopy, including the reduced risk of complications due to cervical dilation and thermal injuries. Since morcellation doesn’t require cervical dilation with Hegar dilators, it minimizes the risk of cervical trauma, uterine perforation, bleeding and infection [19].

In addition, morcellation does not use electrosurgical energy, thus eliminating thermal risks to nearby organs, such as intestine, bladder, and blood vessels, which can occur during resectoscopic procedures using monopolar or bipolar electrosurgical energy. Instead, mechanical tissue removal prevents potential burns and vascular damage, making morcellation a less-invasive procedure with fewer associated risks and enhancing the overall safety profile [17–20].

Another important benefit of morcellation is the preservation of tissue integrity, which improves the quality of pathological analysis. In traditional resectoscopy, electrosurgical energy can cause thermal artifacts that interfere with accurate histological examination. Mechanical morcellation keeps samples intact, allowing pathologists to perform more reliable diagnoses without the interference of heat-induced distortion [21–23].

It is possible to use the 5 mm mini-resector, thanks to its small diameter, makes it possible to avoid cervical dilatation with the associated risks of perforation. Obviously, the risks associated with the use of electricity remain, making it necessary to be more careful when handling the instrument with the patient awake. It can be used in cases of uterine malformations, particularly uterine septa and myomas, as well as in cases of fundic endometrial polyps.

The risk of perforation using the morcellator is reduced as it is a surgical approach under vision, without blind cervical dilatation. The same risk is superimposable with the use of the mini-resector.

Outpatient "see-and-treat" morcellation also presents clear economic advantages. By eliminating the need for the pre-hospitalization visit and operating room costs, the procedure is more cost-effective. Not to be underestimated is the economic impact in terms of abstention from work for the patient, who in a day-hospital setting is forced to give up three working days versus a single absence, with consequences also for the employer. The overall efficiency of the technique, combining diagnosis and treatment in one visit, helps avoid additional hospital admissions and reduces the use of operating room resources. This time-saving aspect of morcellation, especially for mild cases of abnormal uterine bleeding (AUB) or small polyps, suggests that outpatient hysteroscopy could become the standard of care, particularly in healthcare systems with high patient volumes and limited resources [24, 25].

Regarding the use of the morcellator in the management of infertile patients, the available data are still insufficient. Certainly, the treatment of adhesion syndromes can be approached very safely as it is a procedure performed under direct vision without the need for cervical dilation [26]. With regard to adenomyosis, on the other hand, the morcellator is useful in detecting indirect signs at the subendometrial level, thus contributing to diagnosis; however, the same cannot be said about treatment as it cannot use monopolar or bipolar energy [27]. As for the treatment of uterine septa, the most common form of uterine malformations, the use of the morcellator remains limited, with resectoscopy still considered the gold standard in these cases [28]. However, for the treatment of incomplete miscarriages, the morcellator has now largely replaced dilation and curettage as direct visualization minimizes the risks associated with the procedure [29].

Despite these advantages, the limitations of morcellation must be considered. For instance, it may be less effective for treating fundal polyps**,** especially those with a broad base or deep attachment to the endometrial tissue, which may be difficult to excise completely using the morcellator. [30] Additionally, the morcellator is not ideal for treating larger fibroids (FIGO 0, 1, 2) or lesions with significant myometrial involvement, particularly in cases of misdiagnosis where fibroids are mistaken for smaller polyps [31]. In such cases, excessive bleeding or incomplete removal may occur. Therefore, careful preoperative diagnosis and assessment are essential to avoid these risks and ensure that the most appropriate technique is chosen [32].

This study clearly shows the non-inferiority of the outpatient method over the day-hospital method in the management of suspected endometrial polyps in patients with AUB. In addition, the ‘see-and-treat’ treatment entails an advantage in terms of operative time, economics in relation to the use of surgical instrumentation and working time for the patient, reduction of the number of hospital admissions, and consequently reduction of waiting lists.

The choice of hysteroscopic technique should remain personalized, factoring in patient tolerance, polyps’ characteristics, and the physician’s experience. For an appropriate choice of technique, the patient's clinical history and the ultrasound characteristics of the endometrial polyp must certainly be considered, e.g., a large lesion located on the uterine fundus will require more time and it would be appropriate to remove it in an operative setting, as well as the case of nulliparous patients with hypothetical stenosis of the cervical canal [33]. Outpatient morcellation, though technically demanding, meets high standards of safety and patient satisfaction and is particularly well-suited for women comfortable with mild procedural discomfort who prefer to avoid anesthesia. Given the non-inferiority demonstrated in this study, outpatient morcellation could be increasingly adopted for appropriate cases, offering a safe, efficient, cost-effective, and patient-centered care.

In our experience, the follow-up was favorable in all patients as no recurrence was evident in any group. However, a longer-term follow-up would be useful in future to highlight any differences between the procedures.

In conclusion, outpatient morcellation provides multiple advantages, including reduced anesthesia risks, faster procedure times and better tissue preservation for pathological analysis. However, careful patient selection and personalized treatment remain the key to optimize outcomes. When used appropriately, this approach can significantly contribute to more efficient and patient-centered care.

## Conclusions

In the context of endometrial pathology, polyps can represent a malignant condition in 0.8%–4.9% of cases, with the risk increasing in the presence of factors, such as age, polyp size, and symptoms like bleeding. Given these risk factors, it is necessary to provide treatment to allow histological evaluation of the lesion and improve the woman’s quality of life by addressing a potential cause of abnormal uterine bleeding (AUB).

Today, it is possible to perform both diagnostic and therapeutic procedures in a single session using smaller hysteroscopic devices, which achieve the same outcomes as conventional resectoscopic hysteroscopy under sedation in a day hospital setting.

This experimental study suggests the use of outpatient hysteroscopic procedures without sedoanalgesia as effective, safe, and well-tolerated by patients. With comparable patient satisfaction and minimal pain, outpatient morcellation provides a feasible “see-and-treat” option that reduces procedural time and time to diagnosis, crucial for cases where AUB could indicate endometrial carcinoma. Rather than requiring three appointments for underlying cause removal, the patient can complete all necessary procedures in a single visit. Additionally, managing the patient at the outpatient level avoids day hospital costs, eliminating hospitalization expenses. Incorporating outpatient hysteroscopy as a first-line approach in cases of AUB with endometrial polyps may enhance patient care and optimize resource utilization. Future operating protocols should focus on expanding the use of outpatient hysteroscopy, prioritizing patient experience, clinical efficiency, and economic sustainability [17].

## Data Availability

Data will be made available to the editors of the journal for review or query upon request by corresponding author.
